# Cost of illness for severe and non-severe diarrhea borne by households in a low-income urban community of Bangladesh: A cross-sectional study

**DOI:** 10.1371/journal.pntd.0009439

**Published:** 2021-06-11

**Authors:** Rebeca Sultana, Stephen P. Luby, Emily S. Gurley, Nadia Ali Rimi, Sayeda Tasnuva Swarna, Jahangir A. M. Khan, Nazmun Nahar, Probir Kumar Ghosh, Sushil Ranjan Howlader, Humayun Kabir, Shifat Khan, Peter Kjær Mackie Jensen

**Affiliations:** 1 Department of Public Health, University of Copenhagen, Copenhagen, Denmark; 2 Institute of Health Economics, University of Dhaka, Dhaka, Bangladesh; 3 icddr,b, Dhaka, Bangladesh; 4 Stanford University, Stanford, California, United States of America; 5 John Hopkins Bloomberg School of Public Health, Baltimore, Maryland, United States of America; 6 Liverpool School of Tropical Medicine, Liverpool, United Kingdom; 7 Swiss Tropical and Public Health Institute, Basel, Switzerland; 8 University of Basel, Basel, Switzerland; Rollins School of Public Health, UNITED STATES

## Abstract

The illness cost borne by households, known as out-of-pocket expenditure, was 74% of the total health expenditure in Bangladesh in 2017. Calculating economic burden of diarrhea of low-income urban community is important to identify potential cost savings strategies and prioritize policy decision to improve the quality of life of this population. This study aimed to estimate cost of illness and monthly percent expenditure borne by households due diarrhea in a low-income urban settlement of Dhaka, Bangladesh. We conducted this study in East Arichpur area of Tongi township in Dhaka, Bangladesh from September 17, 2015 to July 26, 2016. We used the World Health Organization (WHO) definition of three or more loose stool in 24 hours to enroll patients and enrolled 106 severe patients and 158 non-severe patients from Tongi General Hospital, local pharmacy and study community. The team enrolled patients between the first to third day of the illness (≤ 72 hours) and continued daily follow-up by phone until recovery. We considered direct and indirect costs to calculate cost-per-episode. We applied the published incidence rate to estimate the annual cost of diarrhea. The estimated average cost of illness for patient with severe diarrhea was US$ 27.39 [95% CI: 24.55, 30.23] (2,147 BDT), 17% of the average monthly income of the households. The average cost of illness for patient with non-severe diarrhea was US$ 6.36 [95% CI: 5.19, 7.55] (499 BDT), 4% of the average monthly income of households. A single diarrheal episode substantially affects financial condition of low-income urban community residents: a severe episode can cost almost equivalent to 4.35 days (17%) and a non-severe episode can cost almost equivalent to 1 day (4%) of household’s income. Preventing diarrhea preserves health and supports financial livelihoods.

## Author summary

The illness cost borne by households, known as out-of-pocket expenditure was 74% of the total health expenditure in Bangladesh in 2017. Most of the studies in Bangladesh that estimated the cost of diarrheal illness collected data from hospital patients and mostly targeted under-five children. Information on economic burden of diarrhea borne by households of low-income urban communities who commonly had ≤ 2 US$ dollar income per capita per day still remained unknown. We conducted this study in East Arichpur area of Tongi township in Dhaka, Bangladesh from September 2015 to July 2016 to estimate cost of illness and monthly percent expenditure borne by households due diarrhea in a low-income urban settlement of Dhaka, Bangladesh. The estimated average cost of illness for patient with severe diarrhea was US$ 27.39 (2,147 BDT) and non-severe diarrhea was US$ 6.36 (499 BDT). A single diarrheal episode substantially affects financial capability of the low-income urban community: a severe episode can cost 4.35 days (17%) and a non-severe episode can cost 1 day (4%) of income of a households. Preventing diarrhea preserves health and supports financial livelihoods.

## Introduction

Diarrhea presents a substantial health burden globally. According to Global Burden Diseases study (GBD), diarrhea remained the eighth leading cause of disability-adjusted life-years (DALY) in all age group and third leading cause DALY in children [1] Unsafe water, unsafe sanitation, and handwashing related DALY was ranked third in low socio-demographic index in 2019 [2].

In Bangladesh in 2016 the estimated DALY due to diarrhea was 1.4 million (1,391,000) [3] and the mortality attributed to exposure to unsafe water and sanitation service was 12 deaths per 100,000 population [4]. A cross sectional study conducted in 2017 reported that 76% of the patients, who reported diarrhea in the preceding month in Dhaka, were slum residents [5]. The World Bank estimation in 2014 reported that 55% of the urban population live in urban slums in Bangladesh [6] and more than 90% of households in urban slums share sanitation facilities and water sources [7]. Despite the vulnerability to many health problems, including diarrheal diseases, the scientific literature on slum health is underdeveloped compared to that on urban health [8]. Sixty-five to ninety-five percent of all diarrhea episodes in low-income areas are mild and moderate cases [9]. These mild and moderate cases are missed in the prevalence and cost estimates due to scarcity of community based diarrheal disease burden studies, as diarrhea prevalence studies are mainly focused on child diarrhea [10,11]. Additionally, the first point-of- care for diarrhea of low-income population is non-professional healthcare providers (80%), not the hospitals [11]. Thus, the low-income urban communities endure a high burden of diarrhea, which has an important economic impact on the household’s economy and on the community.

The illness cost borne by households, known as out-of-pocket expenditure, was 74% of the total health expenditure in Bangladesh in 2017 [12]. In 2017, 3.3% of the population was pushed into poverty due to out-of-pocket healthcare expenditure in Bangladesh [13]. The high out-of-pocket expenditure not only increases poverty but also can increase avoidance of seeking treatment due to the high expenditure [14], which potentially exposes people to higher risk of mortality.

The broader estimation of cost may not be pertinent to the low-income urban communities as the household cost of diarrhea varies considerably due to diverse geographical location [14,15] including rural [10] and urban [16], socio-economic condition, per capita income, care seeking practices and health care facilities [17]. A study on household cost of diarrhea among <5 children in three Asian countries reported that in 2011 the cost per episode was US$ 1.82 in Bangladesh, US$ 3.33 in India and US$ 6.47 in Pakistan [14]. Most of the studies that estimated the cost of diarrheal illness collected data from hospitalized patients [17–21] and mostly targeted children aged less than five [10,14,15]. However, many of these studies [10,14,15,19,20] did not estimate the cost of patients of all ages. Many of these studies also did not estimate the cost of patients who visited a pharmacy or followed self treatment at home [17–21]. A recent national study in Bangladesh showed that 46% of people did not seek care from hospitals for childhood diarrhea (23% sought no care and 23% sought care from pharmacy) [22]. Thus, the magnitude of this cost to the household income of low-income urban communities who commonly had ≤ 2 US$ dollar income per capita per day still remained unknown [6,23]. A comprehensive study on household cost of diarrhea of low income communities in all age group including gender, diverse care seeking practices (i.e., from hospital, from pharmacy, self-treatment at home), and household percent expenditure for an episode would better inform the health policies of the government to prioritize and improve health services for the low-income and disadvantages population to ensure equity. This information could also be useful for evaluating potential interventions to reduce health burden either through vaccination [24], improvement of water and sanitation [25] or other interventions [26]. Therefore, this study aimed to estimate cost of illness and monthly percent expenditure borne by a household due to severe and non-severe diarrhea in a low-income urban settlement of Dhaka, Bangladesh.

## Methods

### Ethics statement

The data collection team obtained written informed consent from all the participants or their guardians (for children) included in this study. The Institution Review Board of icddr,b named as "Ethical Review Committee of icddr,b" approved the study protocol.

### Study sites

We conducted this study from September 17, 2015 to July 26, 2016 in East Arichpur area of Tongi Township in Dhaka, Bangladesh. People living in this area were at high risk for diarrheal and waterborne diseases [27,28]. East Arichpur is predominantly a low-income urban community with 13,876 households and approximate 55,504 population living within <1 km^2^ [29]. In East Arichpur, people frequently lived in a setting where a nuclear family shared one room and multiple families/households shared a common yard (locally known as compound), toilet/stove/ water source [29]. Communities living in such density with limited water and sanitation facilities are usually considered as a low income urban community [30].

We selected the ’Shaheed Ahsanullah Master 250-bed General Hospital, Tongi’ as the hospital site and East Arichpur area as the community site. This hospital was the only government primary health facility for the population of Tongi Township. It has a separate allocated place to treat admitted patient of diarrhea. The total outdoor patient department visits to this hospital in 2016 were 175,788 [31].

### Definition of severe and non-severe diarrhea

We used the World Health Organization (WHO) definition of ’diarrhea’ that includes three or more loose stools within last 24 hours [32]. To classify the severe diarrhea patient, we considered physicians’ perception of hospital, since the physicians provides IV saline to the patients with ’some’ or ’severe’ dehydration as defined in WHO guideline [32]. Using physicians’ perception to identify patients was also used in other studies [9,33]. We classified patients as ’severe’ if they received intravenous saline and/or were admitted in the hospital to receive rehydration treatment [32]. We considered the patient who had three or more loose stools in 24 hours, followed self treatment at home or treatment sought from pharmacy and did not use intravenous fluid, as a ’non-severe’.

### Subject enrolment

**Fig 1:**
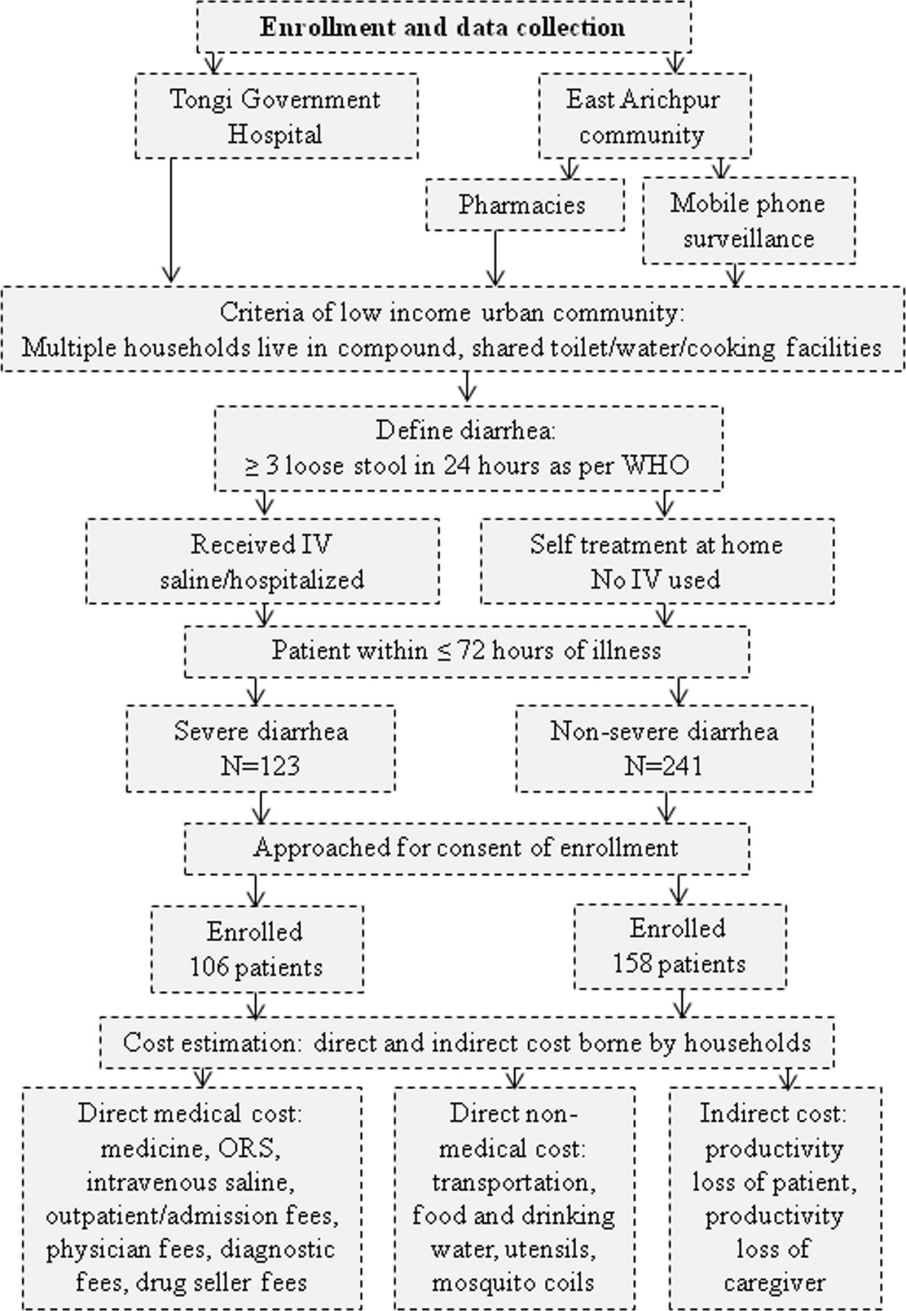
Participant enrollment process and cost data collection of the study conducted in Tongi Township in Dhaka, Bangladesh from September 2015 to June 2016.

The study participants were diarrhea patients living in low-income urban communities (Fig 1). The research team prospectively enrolled patients between their first to third day of the illness (≤ 72 hours) and continued daily follow-up with them until their recovery (i.e., return to their daily normal activities). The researcher retrospectively collected costs that were incurred prior to the first day of interview.

The team enrolled hospitalized diarrhea patients. A researcher trained in quantitative methods visited the Tongi General Hospital once or twice a day to identify hospitalized diarrhea patients who fulfilled the enrollment criteria (Fig 1).

For the community site, the team enrolled patients from local pharmacies and from a community based mobile phone surveillance of diarrhea of East Arichpur, Tongi [23] (Fig 1). The team selected 35 local pharmacies out of 52 based on willingness of the drug sellers to participate in this study. The team informed the drug sellers working at the pharmacies about the diarrhea definition (i.e., three or more loose stools in 24 hours). If any customer bought oral rehydration solution (ORS), other medicine and/or intravenous saline, the drug seller asked the customer about the illness of the patient to know if the patient had diarrhea. If the customer reported about a diarrhea patient, then the drug seller sought willingness of the customer to participate in our study and based on customers’ consent the sellers collected the phone number or address of the customer and informed the research team over phone. The research team communicated with customers within 24 hours of receiving the information from drug seller and enrolled the patient if s/he fulfilled the enrollment criteria (Fig 1). Afterward, a researcher visited the patient’s house to conduct a face-to-face interview.

A pre-paid mobile phone based surveillance was ongoing in East Arichpur as part of a longitudinal study of diarrhea incidence to collect data from 419 low-income households [23]. We used this call center to enroll the patients if s/he fulfilled the enrollment criteria (Fig 1).

The team conducted interviews with patients and/or adult household members who were informed about the cost incurred during the entire illness episode. The team first conducted face-to-face interviews and then followed them up through phone interview to collect cost information on each day of illness. The team also collected data on coping with cost of illness from the respondents. The hospitalized patients were also followed up to collect cost data after returning home until recovery.

### Cost estimates

To collect and estimate the cost of diarrheal illness incurred by the study households, we used WHO guidelines of economic burden estimation of diarrhea [34]. Household cost of diarrhea was defined as out-of-pocket payments made by the households during the illness episode for treatment of diarrhea patient (direct medical and direct non-medical) and opportunity/indirect costs was for the time used by patients and/or caregivers [35] (Fig 1). We considered loss of productivity to estimate indirect cost. For paid workers, the daily income loss was estimated based on the reported daily wages of the patients and caregivers. The value of daily productivity for unemployed individual was assumed based on age-specific wage and was divided into three groups: adult, teenager (aged 11 to 17) and children aged 5 to 10 [18,36]. Although the children were not involved in income generating activities but they lost school days and thus we estimated cost of children following other studies [18,36]. Similarly, we also estimated average daily wages for unemployed individuals following other studies [18,36] and considered one-half of average patient’s daily wages (who were involved in income generating activities) for adults and teenagers (working at own home, student), and one-quarter of patient’s daily wages for children [21,36].

### Sample size calculation

Based on a pilot study, we assumed the mean cost per episode for severe diarrhea was BDT 1,406 (US$ 17.93) and standard deviation (SD) was 727 (US$ 9.27). The mean cost per episode for non-severe diarrhea was BDT 450 (US$ 5.73) and SD was 269 (US$ 3.43). We considered 10% precision as recommended by WHO [34] with 95% confidence interval for sample size calculation. Therefore, to estimate the cost of diarrhea per episode for each group, we needed to enroll 106 severe and 158 non-severe diarrhea patients [34].

### Data analysis

We used descriptive statistics (mean, standard deviation [SD], median) to present the cost. We also calculated confidence interval for the total illness cost of the patients. We calculated the cost of severe and non-severe diarrhea by age group, illness duration and gender. The percent expenditure of household income for a diarrhea episode was calculated by total expenditure for a diarrhea episode divided by monthly household income and multiplied by 100 for a patient [20]. All costs were collected in Bangladesh Taka (BDT) and converted to US dollars. The conversion rate was adjusted to reference year 2016 and the exchange rate was US$ 1 = 78.4 BDT [37].

To estimate the annual cost of diarrhea in East Arichpur, we applied the published incidence rate of mobile based surveillance of East Arichpur from the longitudinal study [23] to estimate the incidence based economic burden borne by household. To estimate the hospital incidence based economic burden, we estimated the number hospital admission of diarrhea patients from East Arichpur in Tongi General Hospital from January to December 2016. We have calculated the population based incidence of severe diarrhea by dividing the total hospitalized diarrhea cases of East Arichpur with the total population of East Arichpur. We used the information of the population from the census data of Sultana et. al. (2019) [29] and the census of Bangladesh Bureau of Statistics (BBS) [38]. We estimated number of non-severe diarrhea cases by multiplying incidence rate from longitudinal study [23] with the total population of East Arichpur.

## Results

The study identified 123 severe and 241 non-severe diarrhea patients and enrolled 106 severe and 158 non-severe patients to collect data on cost per episode of diarrhea borne by households (Fig 1). Forty percent of the severe patients were garment or other factory workers; 27% of the non-severe patients were children ≤ 5 years (Table 1). The median duration of illness was three days for severe and two days non-severe diarrhea patients (Table 1).

**Table 1:** Education and occupation of the severe and non-severe diarrhea patients of Tongi Township in Dhaka, Bangladesh from September 2015 to June 2016.

Characteristics	Severe patients		Non-severe patients	
	N = 106	(%)	N = 158	(%)
Age		Or [SD]		Or [SD]
Mean age [standard deviation (SD)]	25	[12.6]	22	[17.8]
Mean monthly income per household (US$) [SD]	190	[104]	199	[84]
**Education**				
No schooling yet	10	(9)	48	(30)
No education	15	(14)	29	(18)
Did not complete primary	26	(25)	32	(20)
Completed primary	34	(32)	37	(23)
Completed secondary	12	(11)	5	(3)
Completed higher secondary	5	(5)	3	(2)
Bachelor and above	4	(4)	4	(3)
**Occupation**				
Not eligible for work (children ≤ 5 years)	10	(9)	45	(27)
Not involved in earning	25	(24)	58	(37)
Garment or other factory workers	42	(40)	17	(11)
Service holders	11	(10)	4	(3)
Day labors	-	-	9	(6)
Mobile vendor	1	(1)	8	(5)
Mechanics	10	(9)	5	(3)
Vehicle driver	3	(3)	-	-
Shopkeepers and small business	2	(2)	6	(4)
Business	-	-	4	(3)
Household help	2	(2)	2	(1)
**Male and female involved in earning**				
Male	43	(41)	41	(26)
Female	28	(26)	14	(9)
**Duration of illness and stool frequency**				
Illness duration in days [SD]	3.2	[1.1]	2.5	[1.0]
(Median)		(3)		(2)
Stool frequency per day/person [SD]	6	[2.0]	4	[1.7]

Ninety-two percent (97/106) of patients with severe diarrhea were admitted to hospital (52 males, 45 females). The nine non-hospitalized severe patients received intravenous fluid infused by the drug sellers of the pharmacies at home. Eight of them were advised by drug seller and one advised by a physician. Sixty-five (34/52) percent of the male and 62% (28/45) of the female stayed in the hospital for ≥ 2 days.

### Cost and percent expenditure for severe and non-severe diarrhea

The average total cost of illness per episode for severe diarrhea was 2,147 BDT (US$ 27.39) and per episode for non-severe diarrhea was 499 BDT (US$ 6.36) (Table 2). Severe diarrhea consisted of 17% of the average monthly household income and non-severe diarrhea consisted of 4% of the average monthly household income of the participants (Table 3). Most of the severe patients paid for intravenous rehydration, outpatient fees, diagnosis, consultant fees and payment for drug sellers’ home visits, whereas only two of the non-severe patients paid for outpatient fees and one of the patient spent money for consultant fees (Table 2). For severe diarrhea, the average direct cost represented 40% of the total illness cost and for non-severe diarrhea, average direct cost represented 26% of the total illness cost.

**Table 2:** Average household cost for severe and non-severe diarrhea of Tongi Township in Dhaka, Bangladesh from September 2015 to June 2016, US$*.

Cost parameter	Severe diarrhea			Non-severe diarrhea		
(Cost presented in US$)	N = 106	Mean	SD^†^	N = 158	Mean	SD^†^
**Direct medical**	106	6.47	4.05	158	1.28	2.02
Medicine	98	2.68	3.20	140	0.95	1.4
Oral rehydration	99	0.46	0.35	154	0.38	0.29
Intravenous rehydration (IV)	96^‡^	3.44	1.40	-	-	-
Admission/ registration fee	-	-	-	-	-	-
Outpatient fee	91	0.13	1.40	2	0.25	-
Diagnostic	1	8.92	-	-	-	-
Physician/Consultant fee	7	3.37	1.50	1	8.92	-
Drug seller home visit	4	0.80	0.31	-	-	-
**Direct non-medical**	106	4.40	5.14	73	0.78	0.64
Transportation	96	1.88	4.37	5	0.4	0.29
Food items	104	2.17	1.66	72	0.78	0.64
Caregiver’s food and other cost	49	0.91	0.75	-	-	-
mosquito coil, soap and others	56	0.28	0.24	-	-	-
Informal payment	-	-	-	-	-	-
**Total direct cost**	106	10.88	6.92	158	1.64	2.2
Patient’s income loss	96	10.61	7.78	65	7.5	5.2
Caregiver’s income loss	114	6.45	4.09	98	2.62	3.99
**Total indirect cost**	106	16.52	11.05	158	4.73	6.32
**Cost of illness of household**	**106**	**27.39**	**14.74**	**158**	**6.36**	**7.2**
**(BDT)**		**(2,147)**			**(499)**	
**[95% CI]**	**[24.55,30.23]**				**[5.19, 7.55]**	

*1 US$ = 78.4 Bangladesh Taka within Jan-Jun 2016.

^†^SD = Standard deviation.

^‡^IV cost was not included for those patients who received free IV from hospital.

All the cost in BDT is included in the S1 Table.

**Table 3:** Percent expenditure of monthly household income for severe and non-severe diarrhea of Tongi Township in Dhaka, Bangladesh from September 2015 to June 2016.

Percent expenditure of monthly household income	Severe diarrhea			Non-severe diarrhea		
	N	Mean	SD*	N	Mean	SD*
Direct medical	106	3.8	2.2	158	0.7	0.9
Direct non-medical	106	2.8	4.7	73	0.4	0.4
Total direct cost	106	6.7	5.6	158	0.9	1.1
Total indirect cost	106	10	6.4	158	2.6	3.9
Cost of illness of household	106	16.6	9.9	158	3.6	4.5

*SD = Standard deviation

### Cost of diarrheal illness by age and sex

The average illness cost was higher among adults than among children both for severe and non-severe diarrhea because of the loss of productivity due to illness (Table 4). The cost of illness was higher for the male patients compared to female patients both for severe and non-severe diarrhea. Among the non-severe patients, cost of food for patients was the common direct non-medical cost and it was higher for male (mean 0.82, SD 0.69, median 0.66 US$) compare to female (mean 0.72, SD 0.57, median 0.51 US$).

**Table 4:** Average household cost of severe and non-severe diarrhea by age, gender and duration of illness of Tongi Township in Dhaka, Bangladesh from September 2015 to June 2016, US$.

		Severe diarrhea				Non-severe diarrhea		
	N	Direct cost	Indirect cost	Total cost	N	Direct cost	Indirect cost	Total cost
		Mean (median)	Mean (median)	Mean (median)		Mean (median)	Mean (median)	Mean (median)
**Age group**								
1–4	10	10.2 (9.3)	10.3 (9.4)	20.5 (22.1)	45	2.4 (2)	2.6 (0.8)	5 (2.7)
5–10	6	9.7 (8.5)	15.8 (16)	25.5 (28)	8	1.4 (0.9)	2.2 (1.4)	3.6 (2.9)
11–17	4	9.6 (7.7)	17.1 (18.3)	26.7 (26.7)	15	0.9 (0.4)	3.5 (1.1)	4.4 (1.8)
18+	86	11 (8.3)	17.2 (14.8)	28.3 (25.5)	90	1.4 (1.1)	6.2 (4.2)	7.6 (5.2)
**Gender**								
Male	56	11.4 (8.2)	17.1 (14.3)	28.4 (24.6)	85	1.8 (0.9)	5.3 (3.1)	7.2 (4.3)
Female	50	10.3 (8.6)	15.9 (14.7)	26.2 (25.8)	73	1.5 (1.2)	4 (0.8)	5.4 (2.8)
**Illness duration**								
1–2 days	29	8.2 (7.1)	13.9 (13.7)	22.2 (22)	81	0.9 (0.7)	3.2 (1.3)	4.1 (3)
3–4 days	66	11.1 (9.2)	15.5 (14.4)	26.6 (25.1)	71	2.3 (1.8)	6 (1.6)	8.3 (4.6)
5–7 days	11	16.6 (15.1)	29.1 (24.7)	45.7 (35.5)	6	3.5 (3.6)	9.2 (6.8)	12.7 (9.9)

All the cost in BDT is included in the S2 Table.

### Use of antibiotics

Reported antibiotic use was more common among the non-severe patients (74%) compared to severe patients (54%) (Table 5). Metronidazole was the most commonly reported antibiotic by both severe (86%) and non-severe (90%) patients.

**Table 5:** Coping strategies and use of antibiotic among severe and non-severe diarrhea patients of Tongi Township in Dhaka, Bangladesh from October 2015 to June 2016.

Characteristics	Severe patients		Non-severe patients	
	N = 106	(%)	N = 158	(%)
Coping with cost of diarrhea				
Reduced food consumption	30	(28)	37	(23)
Savings	24	(23)	31	(19)
Borrowing money	18	(17)	11	(7)
Work for extra hours to get extra money	16	(15)	14	(9)
Send less amount to the family at homeland	10	(9)	4	(3)
Gift or helped by others	4	(4)	1	(1)
Cost paid by office	4	(4)	-	-
The amount of cost was small	-	-	60	(38)
**Medicine and antibiotic use**				
Did not know the name of medicine	26	(25)	14	(9)
Male	12	(11)	10	(6)
Female	14	(13)	4	(3)
Did not use medicine^†^	19	(18)	18	(11)
Male	5	(5)	10	(6)
Female	14	(13)	8	(5)
Did not use antibiotic	4	(4)	9	(6)
Male	3	(3)	5	(3)
Female	1	(1)	4	(3)
Used antibiotic	57	(54)	117	(74)
***Name of the antibiotic use***	***n = 57***		***n = 117***	
Metronidazole	24	(42)	58	(50)
Ciprofloxacin	8	(14)	12	(10)
Metronidazole and Ciprofloxacin	25	(44)	47	(40)
***Antibiotic use by sex***				
Male	36	(63)	60	(51)
Female	21	(37)	57	(49)
**Cost of medicine when antibiotic used**		**in BDT**	**in BDT**	
Average medicine cost [SD] (median)	231	[293]	83	[122]
		(134)		(40)

*SD = Standard deviation.

^†^ Patient who did not take any medicine except intravenous saline or ORS.

### Coping with cost of illness

Patients of both severe and non-severe diarrhea reported that they reduced food consumption to cope with the cost of diarrhea (Table 5). The non-severe patient’s respondent commonly reported that the amount of cost of illness was small and did not require additional coping effort. The respondents of both severe and non-severe diarrhea also reported that to return the borrowed money, they reduced food consumption and/or worked for extra hours.

### Annual cost of diarrhea borne by households of East Arichpur

A total of 232 diarrhea cases were admitted in the hospital from January to December 2016 and the population based incidence rate of hospitalization for diarrhea was 4 per 1000 person years (95% CI: 3.7–4.8). The annual cost for severe cases of East Arichpur was 6,355 US$. The incidence for the community cases identified through mobile phone surveillance from August 2014 to June 2015 was 0.160 (95% CI: 0.13–0.19) per person year [23]. The estimated community based diarrhea burden in the population of East Arichpur would be annually 8,881 cases. Thus, 3% (232/8,881) of estimated cases were visited to hospital. Therefore, the estimated annual cost for non-severe cases of East Arichpur was 55,008 US$.

## Discussion

The low-income urban community spent a notable amount of money due to an episode of severe diarrhea that led the household to compromise their regular food intake, savings and borrow money as coping strategies. Although the average cost of non-severe diarrhea at household level was low as US$ 6.36 with a percent expenditure of 4%, the estimated incidence based economic burden of the community was high (US$ 55,008) demonstrating economic loss due to non-severe diarrhea within this community. Averting one episode of severe diarrhea can save 4.35 days (17%) of monthly income and one episode of non-severe diarrhea can save 1 day (4%) of income of a household in the low-income communities.

The cost of some preventive initiatives for diarrheal diseases identified in previous studies can be comparable with the cost of one episode of severe and non-severe diarrhea. For example, a household-based intervention on drinking water chlorination can cost US$ 0.66 and filtration can cost US$ 3.03 person per year [25], and such intervention to improve drinking water quality are estimated to reduce diarrhea by 30% [39]. The other example could be to promote a dedicated tools Sani-Scoop (a mini hoe) (cost US$ 1–1.5) [40] and potty (cost US$ 1–6) for removing child and animal feces from the household premises that was used in the WASH benefit trials [40–42]. Although the WASH benefit trials studies suggest no effect of WASH in child growth, the re-analyzed of control group data showed a relationship with length-for-age Z (LAZ) score at 18–24 months children and improve sanitation [41]. Furthermore, the WHO estimated that the unit cost for house connection of water supply in urban area is US$ 5.8 (US$ 2.8 annual capital cost and US$ 2.9 annual recurrent cost) and the unit cost for sewerage connection is US$ 23.1 (US$ 7.9 annual capital cost and US$ 15.2 annual recurrent cost) in Bangladesh [43]. Employing community health workers to provide health education, including messages on diarrhea prevention, via household visits could have a two-fold benefit. First, it can be a useful approach to ensure equitable access to health service in achieving universal health coverage by the government. Second, it can reduce total costs, because the cost of a community health worker is estimated to be $6 per person covered per year in Bangladesh [44]. Preventing diarrhea could cover much of these expenses.

The cost of illness for female patients was lower than males as found in other studies [14,18]. The cost among males for severe diarrhea was high, which might be explained by the higher hospitalization, longer hospital stay and higher use of antibiotics for male patients, found in this study compared to female patients. The increased indirect cost among male patients for non-severe diarrhea was likely due to more males being involved in income generating activities than females. The increased direct cost among male patients for non-severe diarrhea might be explained by direct non-medicine cost, which was due to food and transportation cost. These findings were consistent within Bangladeshi context, since gender discrimination exists in Bangladeshi society for health and nutrition related behavior for females [45]. In Bangladesh, male household members are usually prioritized for health care investments [45–47]. The cost of illness was increased by the increase of age of patients and duration of illness, which might be explained by the wage loss of patients and caregivers.

Our study found higher antibiotic use among the non-severe patients than the severe patients, which might have resulted from self-medication [48], and/or advice from the drug sellers of the pharmacy as the first point-of-care [11], common practices for diarrheal illness in Bangladesh. Similar to another study [23,48], metronidazole was the most commonly used antibiotic by our study participants. The WHO guidelines for treating diarrhea explicitly discourage the use of antibiotics to treat acute diarrhea unless in severe cases suspected as cholera because of its ineffectiveness in the majority of cases mild to moderate diarrhea with non-bacterial aetiology [32]. The use of metronidazole is usually recommended for prolonged diarrhea [49] and diarrhea caused by ameobiasis and giardiasis.

Reduction of food consumption was a consequence to cope with the additional expenditure due to illness for the study participants. Compromising food consumption can instigate undernourishment and malnutrition and consequently perpetuate stunning, wasting, infection and disease [50].

A study in a low-income urban community in India among all ages reported that 7–8% of the monthly income spent for an episode of diarrhea [16], whereas our study found 17% of the monthly income spent for severe and 4% of the monthly income spent for non-severe diarrhea. The difference of estimated economic burden and percent expenditure identified in our study from the study of India might be due to different time period and geographical settings, care seeking practices and most importantly might be due to method of cost data collection. In our study, we attempted to collect cost data within a shorter recall period (i.e., 48 hours of illness), whereas most of the studies attempted to collect cost data of diarrheal within 7 to 15 days of illness [10,16,18]. Therefore, participants of those studies might have over-estimated the cost incurred or missed to report some of the additional cost due to the longer recall period.

We estimated that 3% of the community diarrhea patients visited health facilities, which is almost in line the study conducted by Halder et al (2017), where 2% of the children with diarrhea admitted to hospital in the preceding year [10]. Hence, most of the studies that estimated the economic burden of hospital patients [17–21] represent only 2–3% of the total diarrhea burden suggesting that existing literature lacked information on economic burden of diarrhea from a large portion of non-severe diarrhea cases hidden within the communities.

Our study has some limitations. Inclusion of only the nearest government hospital for severe cases might underestimate cost, since we did not estimate the cost of patients who visited other private facilities. Furthermore, we were able to enroll few children as severe patients, which might be due to the different care seeking practices for children in Bangladesh. However, Tongi General Hospital is a top preference for the residents of Arichpur who sought care from hospitals, as half (160/315) of the diarrhea cases of Arichpur were identified from this hospital between April to October 2013 [28]. For the incidence based cost estimation, we considered all the diarrhea cases of our hospital study sites and were unable to include cases only from low-income households due to unavailability of this information, which might be an overestimation. However, our findings on proportion of diarrhea cases that visited hospital were similar with the findings conducted by Halder et al (2017) [10]. The findings from Arichpur might not be generalizable across Bangladesh but the conditions are fairly typical of low income urban settings in Bangladesh. With the paucity of available literature on this common problem, this study provides some useful systematically collected information.

Our study provides an understanding about the household economic burden of a low-income urban community by estimating cost and percent expenditure of both severe and non-severe diarrhea among the patients of all age group from hospitals and communities, and gender specific differences of cost. Efforts to prevent diarrhea are important for improving health of low-income communities and saving money. The Government of Bangladesh introduced a pilot health-financing scheme for the below-poverty population, with a future plan to cover all low-income, disadvantages and marginalized population, to ensure equitable access on health service to achieve universal health coverage [51]. Interventions such as improving hand washing practices and the quality of water can reduce the frequency of diarrheal episodes as well as reduce the financial burden to the low- income community. Community health workers can be engaged to raise hygiene awareness. Our findings can also inform the governments’ health care financing scheme for low income people to plan health care to treat and prevent diarrhea as well as provide financial support during illness episodes.

## Supporting information

S1 Table Average household cost for severe and non-severe diarrhea of Tongi Township in Dhaka, Bangladesh from September 2015 to June 2016, BDT. (DOCX)

S2 Table Average household cost of severe and non-severe diarrhea by age, gender and duration of illness of Tongi Township in Dhaka, Bangladesh from September 2015 to June 2016, BDT. (DOCX)
