# Supplementary material for: Cost of illness for severe and non-severe diarrhea borne by households in a low-income urban community of Bangladesh: A cross-sectional study
Source: PLoS Negl Trop Dis. 2021 Jun 11;15(6):e0009439. doi: 10.1371/journal.pntd.0009439 (PMC8221788; doi:10.1371/journal.pntd.0009439)
Supplement: S1 Table — (DOCX) [file pntd.0009439.s001.docx]

**S1 Table. Average household cost for severe and non-severe diarrhea of Tongi Township in Dhaka, Bangladesh from September 2015 to June 2016, BDT**

| **Cost parameter** | **Severe diarrhea** | | | | **Non-severe diarrhea** | | | |
| --- | --- | --- | --- | --- | --- | --- | --- | --- |
|  | **N=106** | **Mean** | **SD**^*^ | **Median** | **N=158** | **Mean** | **SD**^*^ | **Median** |
| **Direct medical** | 106 | 508 | 318 | 398 | 158 | 101 | 159 | 52 |
| Medicine | 98 | 210 | 251 | 113 | 140 | 75 | 114 | 38 |
| Oral rehydration | 99 | 36 | 28 | 30 | 154 | 30 | 23 | 24 |
| Intravenous rehydration (IV) | 96^†^ | 270 | 110 | 260 | - | - | - | - |
| Admission/ registration fee | - | - | - | - | - | - | - | - |
| Outpatient fee | 91 | 10 | 0.2 | 10 | 2 | 20 | - | - |
| Diagnostic | 1 | 700 | - | - | - | - | - |  |
| Physician/Consultant fee | 7 | 264 | 118 | 200 | 1 | 700 | - | - |
| Drug seller home visit | 4 | 63 | 25 | 50 | - | - | - | - |
| **Direct non-medical** | 106 | 345 | 404 | 264 | 73 | 61 | 50 | 50 |
| Transportation | 96 | 148 | 343 | 80 | 5 | 32 | 23 | 40 |
| Food items | 104 | 171 | 131 | 140 | 72 | 60 | 50 | 50 |
| Caregiver's food & other cost | 49 | 71 | 59 | 50 | - | - | - | - |
| Utensils (mosquito coil, soap and others) | 56 | 22 | 19 | 19 | - | - | - | - |
| Informal payment | - | - | - | - | - | - | - | - |
| **Total direct cost** | 106 | 853 | 543 | 665 | 158 | 128 | 173 | 85 |
| Patient’s income loss | 96 | 832 | 610 | 760 | 65 | 591 | 409 | 500 |
| Caregiver’s income loss | 114 | 506 | 321 | 450 | 98 | 205 | 235 | 83 |
| **Total indirect cost** | 106 | 1295 | 867 | 1149 | 158 | 371 | 495 | 117 |
| **Cost of illness of household** | **106** | **2147** | **1156** | **1981** | **158** | **499** | **590** | **259** |
| [95% CI] | [1925, 2370] | | |  |  | [407, 592] | | |

^*^SD = Standard deviation.

^†^IV cost was not included for those patients who received free IV from hospital.
