# Supplementary material for: Cost of illness for severe and non-severe diarrhea borne by households in a low-income urban community of Bangladesh: A cross-sectional study
Source: PLoS Negl Trop Dis. 2021 Jun 11;15(6):e0009439. doi: 10.1371/journal.pntd.0009439 (PMC8221788; doi:10.1371/journal.pntd.0009439)
Supplement: S2 Table — (DOCX) [file pntd.0009439.s002.docx]

**S2 Table. Average household cost of severe and non-severe diarrhea by age, gender and duration of illness of Tongi Township in Dhaka, Bangladesh from September 2015 to June 2016, BDT**

|  | **Severe diarrhea** | | | | **Non-severe diarrhea** | | | |
| --- | --- | --- | --- | --- | --- | --- | --- | --- |
|  | **N** | **Direct cost** | **Indirect cost** | **Total cost** | **N** | **Direct cost** | **Indirect cost** | **Total cost** |
| **Age group** |  | Mean  (median) | Mean  (median) | Mean  (median) |  | Mean  (median) | Mean  (median) | Mean  (median) |
| 1-4 | 10 | 799 (728) | 807 (739) | 1606 (1734) | 45 | 185 (160) | 208  (62) | 393 (215) |
| 5-10 | 6 | 762 (665) | 1239 (1253) | 2000 (2197) | 8 | 113  (73) | 173 (110) | 285 (226) |
| 11-17 | 4 | 753 (608) | 1344 (1438) | 2096 (2098) | 15 | 72  (30) | 273  (83) | 346 (143) |
| 18+ | 86 | 869 (651) | 1354 (1161) | 2223 (1997) | 90 | 111  (85) | 486 (330) | 597 (412) |
| **Gender** |  |  |  |  |  |  |  |  |
| Male | 56 | 893 (645) | 1338 (1127) | 2231 (1933) | 85 | 140  (72) | 422 (248) | 562 (342) |
| Female | 50 | 806 (674) | 1248 (1152) | 2053 (2022) | 73 | 115  (94) | 311  (62) | 426 (224) |
| **Illness duration** | |  |  |  |  |  |  |  |
| 1-2 days | 29 | 645 (560) | 1093 (1078) | 1738 (1728) | 81 | 72  (52) | 254 (100) | 326 (238) |
| 3-4 days | 66 | 868 (717) | 1220 (1132) | 2088 (1970) | 71 | 181 (140) | 474 (124) | 655 (361) |
| 5-7 days | 11 | 1300 (1185) | 2281 (1940) | 3581 (2783) | 6 | 278 (282) | 718 (536) | 996 (777) |
